# An Unusual Timing of Amoxicillin-Induced IgA Vasculitis in an Elderly Patient

**DOI:** 10.7759/cureus.15757

**Published:** 2021-06-19

**Authors:** Emmanuel Fohle, Sean Montgomery, Joseph Murat, Rachel Ness, Tze Shien Lo

**Affiliations:** 1 Internal Medicine, University of North Dakota, Fargo, USA; 2 Internal Medicine, University of North Dakota School of Medicine and Health Sciences, Grand Forks, USA; 3 Internal Medicine, Veterans Affairs Medical Center, Fargo, USA; 4 Dermatology, Fargo Center for Dermatology, Fargo, USA; 5 Infectious Disease, Veterans Affairs Medical Center, Fargo, USA

**Keywords:** infection, amoxicillin, iga vasculitis, henoch-schönlein purpura, palpable purpura

## Abstract

Immunoglobulin A vasculitis is a small vessel vasculitis which is usually common in the pediatric group. It is rare in adult population but more severe than in children. Proposed triggers include infections, malignancy and medications. For most part, the association is made when immunoglobulin A vasculitis develops within two weeks after starting the implicated medication. A 66-year-old male who was treated with amoxicillin/clavulanate for presumed right fourth toe infection but returned to the emergency department 48 hours later with palpable purpura of lower limbs, arthralgia with swollen hands and colicky abdominal pain with nausea. Abdominal computed tomography (CT) scan showed mildly dilated small bowel. Skin biopsies showed leukocytoclastic vasculitis with IgA deposit on immunofluorescence. The patient was treated with a short course of steroid and the rash was significantly reduced during subsequent follow-up. Although amoxicillin/clavulanate is widely prescribed, clinicians need to be aware of this risk and immediately stop it if signs of drug-induced vasculitis develop.

## Introduction

Immunoglobulin A (IgA) vasculitis, formerly known as Henoch-Schönlein purpura (HSP), is a systemic vasculitis that primarily involves small vessels. It most commonly develops in children, with a peak incidence between the ages of 4 and 6 but may be associated with more severe disease in adults [1-3]. Clinical manifestations classically include palpable purpura, non-migratory arthralgia, abdominal pain, and glomerulonephritis, although it can less commonly involve other organ systems as well. Proposed triggers include infections, malignancy and medications which for most part the association is made when IgA vasculitis develops within two weeks after starting the implicated medication [4]. Here, we present a case of IgA vasculitis that developed 48 hours after the patient was treated with amoxicillin/clavulanate.

## Case presentation

A 66-year-old male with medical history of chronic kidney disease (CKD) stage III, type II diabetes complicated with diabetic neuropathy status post-right 5th toe amputation few years ago presented to the emergency department with discharge from the 4th toe ulcer. He developed symptoms of a pruritic rash involving the limbs, arthralgia, and severe diffuse abdominal pain associated with nausea and vomiting 48 hours after starting oral amoxicillin/clavulanate. Review of systems was otherwise unremarkable.

**Figure 1:**
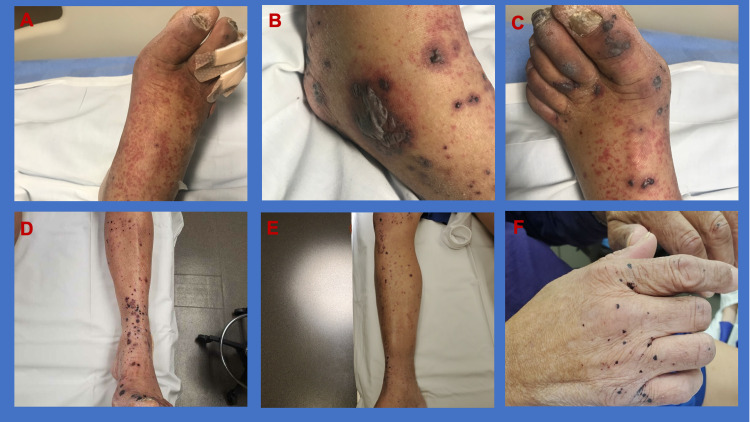
(A-C) Palpable purpura in lower extremities at the time of admission; (D-F) Lower extremities and hands post steroid therapy.

On admission, the patient had a temperature of 36.2°C (97.2°F), heart rate 81 bmp, blood pressure 161/84 mmHg, respiratory rate of 16 breaths/min on room air with SpO2 of 96%. On physical examination, there was symmetric distribution of palpable purpura (Figure 1, Panels A-C) involving both lower extremities with 3mm ulcer on dorsal surface of the 4th right toe but no erythema. His hands were swollen and had limited range of motion. In addition, he had some tenderness with abdominal palpation but no guarding. Laboratory tests are summarized below (Table 1). X-ray of the right foot showed no foreign bodies, soft tissue swelling or osteomyelitis. Abdominal computed tomography (CT) scan showed mildly dilated small bowel.

**Table 1:** Initial laboratory studies at admission

	Admission	Reference range
Hemoglobin	13.3	13.5-17.5 g/dL
Red blood cell	4.66	4.5-6.1 M/cmm
White blood cell	12.5	4.8-10.8 K/cmm
Platelet	247	150-450 K/cmm
Absolute neutrophils	10.7	2-8 K/cmm
Absolute lymphocytes	1.1	1-4 K/cmm
Blood glucose	314	74-109 mg/dL
Sodium	130	136-145 meq/L
Potassium	3.9	3.5-5.0 meq/L
Chloride	93	98-107 meq/L
Bicarbonate	27	22-29 meq/L
Blood urea nitrogen	34	8-23 mg/dL
Creatinine	1.3	0.7-1.2 mg/dL
Calcium	8.7	8.8-10.2 mg/dL
Bilirubin total	0.6	0-1.2 mg/dL
Alkaline phosphatase	73	40-129 U/L
Alanine aminotransferase	8	0-41 U/L
Aspartate transaminase	8	0-40 U/L
C-reactive protein	65	<5 mg/L
Lactate dehydrogenase	126	135-225 u/L

**Figure 2:**
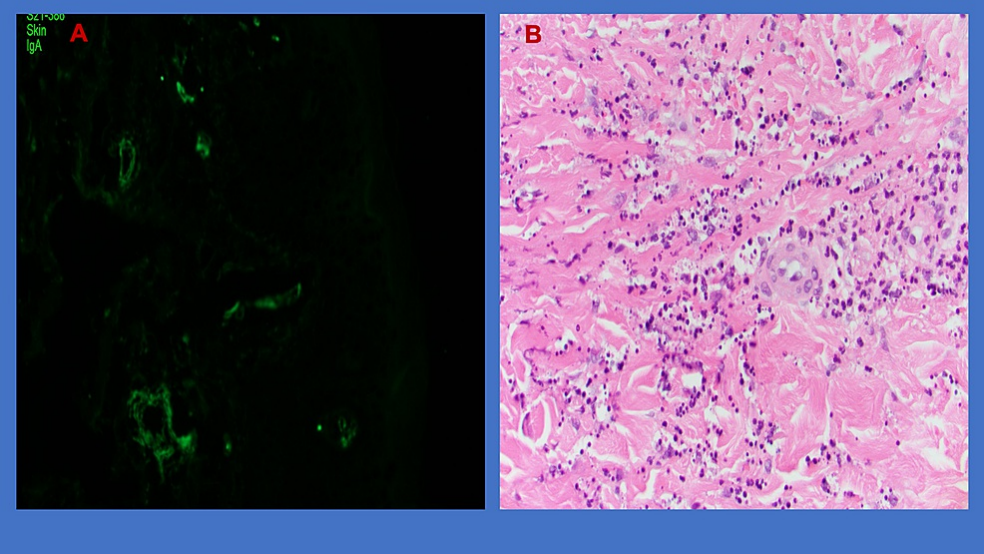
(A) Positive IgA immunofluorescence, (B) fibrinoid necrosis with fragmented neutrophils.

There was a high index of suspicion for IgA vasculitis given the triad of clinical manifestations (palpable purpura, arthralgia and abdominal pain). Steroid was not started because this is often self-limiting. However, on day 3 of hospitalization, he developed non-bloody diarrhea and the rash quickly progressed proximally from both feet to thighs and upper extremities including palms, chest and back. Polymerase chain reaction testing for stool Clostridioides difficile toxin gene was negative. Likewise, blood cultures, urine analysis, serology for hepatitis B virus, hepatitis C virus, human immunodeficiency virus were all negative. Immunologic study including antinuclear antibodies, antineutrophil cytoplasmic antibodies, cryoglobulins and immunoglobulin were all within normal range. Skin biopsies showed leukocytoclastic vasculitis with IgA deposit on immunofluorescence (Figure 2). He was started on a short course of oral prednisone with significant improvement of symptoms and discharged from the hospital on day 5. During subsequent follow-up, his rash significantly receded and the swelling of his hands resolved (Figure 1, Panels D-F).

## Discussion

Cases of antibiotic-induced IgA vasculitis have rarely been reported across all the different classes of antibiotics. Hypersensitivity reactions to penicillin typically present as urticaria and maculopapular rash and is widely reported, but rarely present as vasculitis [5]. Typically, IgA vasculitis would be suspected if the medication was started within a period of two weeks [4]. In this paper, we present a case of a 66-year-old male who developed palpable purpura within 48 hours after being treated with amoxicillin/clavulanate. He was not on any cytochrome p450 inhibitors. Infectious causes were unlikely as our patient displayed no symptoms indicating an infection. The evolution of IgA vasculitis was strongly linked with amoxicillin/clavulanate as the rash erupted 48 hours after starting it. In addition, the patient presented with arthralgia and colicky abdominal pain, which is classical presentation of IgA vasculitis.

IgA vasculitis is a systemic immune complex mediated, small vessel vasculitis most predominately in children. It is very rare in adults with an incidence of 0.8 out of 100,000 adults/year but often severe especially if there is renal involvement with 5% of the cases progress to end stage renal disease requiring dialysis [6,7]. IgA vasculitis typically involves the skin, gut, the joints and glomeruli [8]. It is postulated that IgA deposition in vessel walls plays a major role in the pathophysiology, although the exact etiology remains unclear. A variety of different triggers have been recognized including infectious agents such as bacteria, viruses and parasites, chemical triggers including a number of different drugs and several other environmental triggers [1]. In addition, several genes, most commonly involving the human leukocyte antigen system, have been identified that cause certain individuals to be more susceptible for disease development [1]. In regards to drug-induced IgA vasculitis, vaccines are the most common trigger representing about 23.6% of those cases [9]. Other groups of drugs suspected to have induced IgA vasculitis include antibiotics, TNF-α blockers, anti-hypertensives, analgesics, anticoagulant, antiplatelet agents, and nonsteroidal anti-inflammatory drugs (NSAIDs) [9]. There are no clear data on the prevalence of drug-induced IgA vasculitis given the assessment of drug causality is challenging as there are many other etiological factors involved, the lack of prospective studies and also no specific laboratory markers to discriminate between drug induced and non-drug induced vasculitis [4,5].

According to a committee of the American College of Rheumatology (ACR), the diagnosis of IgA vasculitis is based on the following four criteria: palpable purpura, abdominal pain, age ≤ 20 years and biopsy showing granulocytes in the walls of small arterioles and/or venules [10]. In 2005, the pediatric consensus criteria were developed by the European League Against Rheumatism (EULAR). The mandatory criterion is palpable purpura or petechiae with lower limb predominance in addition to one of the followings: abdominal pain, arthralgia, proteinuria or hematuria, leukocytoclastic vasculitis or proliferative glomerulonephritis with predominant IgA deposition [11].

He was diagnosed with IgA based on both the ACR and EULAR criteria. He exhibited these symptoms 48 hours after being treated with amoxicillin/clavulanate rather than the typical chronological window of 7-10 days, which made this case a rare one [11,12]. He was started on short course of steroid with marked improvement.

## Conclusions

Although amoxicillin/clavulanate is a widely prescribed antibiotic, clinicians should be aware of the rare association of this drug and IgA vasculitis especially that such a condition might deteriorate in a short period of time and how quickly this can occur even outside the age group. Early recognition, prompt discontinuation of the suspected drug and appropriate therapy can limit organ damage.
